# An Unusual Initial Presentation of Lupus Nephritis as a Renal Mass

**DOI:** 10.1155/2015/231974

**Published:** 2015-01-08

**Authors:** Remi Goupil, Annie-Claire Nadeau-Fredette, Virginie Royal, Alexandre Dugas, Jean-Philippe Lafrance

**Affiliations:** ^1^Nephrology Division, Hôpital Maisonneuve-Rosemont, Montreal, QC, Canada H1T 2M4; ^2^Pathology Department, Hôpital Maisonneuve-Rosemont, Montreal, QC, Canada H1T 2M4; ^3^Radiology Division, Hôpital Maisonneuve-Rosemont, Montreal, QC, Canada H1T 2M4; ^4^Medicine Department, Universite de Montreal, Montreal, QC, Canada H3T 1J4; ^5^Centre de Recherche Hôpital Maisonneuve-Rosemont, Montreal, QC, Canada H1T 2M4

## Abstract

Lupus nephritis is a frequent manifestation of systemic lupus erythematous. Lupus nephritis usually presents with abnormal urinalysis, proteinuria, and/or renal insufficiency. We report a case of a 48-year-old woman who underwent partial nephrectomy for a fortuitously discovered solid enhancing left kidney mass. No neoplastic cells were found in the biopsy specimen; however, the pathology findings were compatible with immune complex glomerulonephritis with a predominantly membranous distribution, a pattern suggestive of lupus nephritis. The mass effect was apparently due to a dense interstitial lymphocytic infiltrate resulting in a pseudotumor. Further investigation revealed microscopic hematuria with a normal kidney function and no significant proteinuria. Antinuclear antibodies were negative, although anti-DNA and anti-SSA/Rho antibodies were positive. A diagnosis of probable silent lupus nephritis was made and the patient was followed up without immunosuppressive treatment. After two years of follow-up, she did not progress to overt disease. To our knowledge, this represents the first case of lupus nephritis with an initial presentation as a renal mass.

## 1. Introduction

Lupus nephritis (LN) carries one of the highest morbidity and mortality risks of systemic lupus erythematous (SLE). Usually, LN will be suspected in the presence of active urine sediment, proteinuria, and/or renal insufficiency and confirmed by kidney biopsy. This report describes an unusual case of LN diagnosed fortuitously after a partial nephrectomy for a solid renal lesion.

## 2. Case Presentation

**Figure 1:**
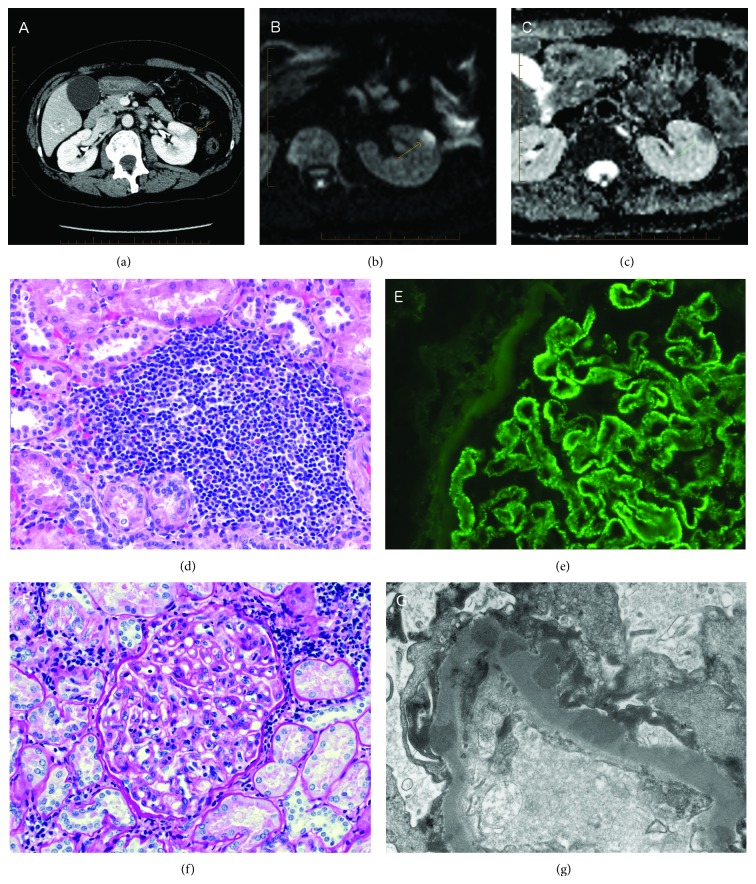
Radiologic and pathologic presentation of the renal lesion. (a) CT scan image showing a homogeneous, enhancing left renal lesion. (b) Diffusion renal magnetic resonance imaging (MRI) image of the same left renal lesion. (c) Apparent diffusion coefficient (ADC) MRI image. (d) Light microscopy with periodic acid-Schiff staining showing a lymphocytic infiltration. (e) Granular positivity of C1q on the glomerular basement membrane by immunofluorescence. (f) Higher magnification of endocapillary hypercellularity. (g) Electronic microscopy showing subepithelial deposits on the glomerular basal membrane.

A 48-year-old woman with abdominal pain was diagnosed with acute cholecystitis based on findings on a computed tomography (CT) scan and ultrasound. The CT scan also showed a 15 × 17 mm homogeneous, enhancing left renal lesion without fat or calcium contents (Figure 1(a)). Magnetic resonance imaging (MRI) revealed a focal left renal iso/mildly hypointense T1 and T2 lesion, with diffusion restriction, and a mild to moderate homogeneous enhancement, without fat content (Figures 1(b) and 1(c)). The differential diagnosis included papillary neoplasm of the kidney, focal lymphomatous infiltration, focal pyelonephritis, and pseudotumoral sarcoidosis. The patient underwent a partial left nephrectomy for a neoplasm suspicion. Pathology evaluation did not show any neoplastic cells but rather a focal and dense lymphocytic infiltrate resulting in a pseudotumor (Figure 1(d)). This interstitial infiltrate was predominantly composed of mature T lymphocyte, with fewer B lymphocytes, and rare plasmocytes, without IgG4 overexpression. Light microscopic evaluation of the glomeruli showed features of membranous glomerulonephritis with diffusely thickened glomerular basal membranes (GBM). Jones' silver stain revealed vacuolisation and spike appearance of the GBM. Few glomeruli showed a focal endocapillary hypercellularity and two presented fibrocellular crescents. Necrosis was not seen. Immunofluorescence microscopy showed granular positivity along the GBM and mesangium for IgG (3+, on 0–3 scale), IgA (3+), IgM (1+), C3 (3+), C1q (2+), Kappa (3+), and Lambda (3+) (Figure 1(e)). Testing for IgG subclasses was uninterpretable, and testing for anti-PLA2R was not performed. Electron microscopy showed numerous subepithelial and mesangial deposits and rare small subendothelial deposits (Figure 1(g)). There were no extraglomerular deposits, and tubuloreticular inclusions were not seen. Thus, a diagnosis of immune complex glomerulonephritis with a predominant membranous distribution was made, a pattern suggestive of lupus nephritis [1].

On further evaluation, the patient denied any symptoms which could have been related to SLE and physical examination was noncontributory. Past personal and familial medical history were noncontributory, without known autoimmune or renal conditions. Laboratory tests were as follows (Table 1): creatinine 60 *μ*mol/L, microscopic hematuria (3–5 red blood cells (RBC) per field on multiple occasions and 6–10 RBC per field once), absence of urinary casts, urine albumin-to-creatinine ratio 7.6 mg/mmol (normal < 3.4), negative antinuclear antibodies (ANA), anti-DNA 424 × 10^3^ IU/L (normal < 55 × 10^3^), anti-SSA/Rho and anti-SSB/La 10.4 and 15.9 IU/L, respectively (normal < 8.0), normal C3, lower limit of normal C4, serum IgG 28.7 g/L (normal 5.5–16.3), IgG4 subclass 0.6 g/L (normal 0.07–0.88), and negative HIV, hepatitis B and C viruses testing.

Considering the absence of major signs of disease activity, the patient was first observed without treatment. During the subsequent 12 months, the patient remained asymptomatic with persistent microscopic hematuria and microalbuminuria (Table 1). In order to clarify prognostic and to determine treatment indication, a right kidney biopsy was performed (11 glomeruli were obtained). The pathology confirmed an immune complex glomerulonephritis with a predominantly membranous distribution, with immunofixation showing deposition of IgG, IgA, IgM, C3, and C1q, but without any signs of focal proliferation. The patient was further observed without immunosuppressive therapy. Eighteen months after initial diagnosis, she reported arthralgia in both hands and was referred to a rheumatologist. She had no physical signs of lupus-related arthritis and, as hematuria and albuminuria had disappeared and both ANA and anti-DNA levels were negative, it was decided to continue observation without treatment.

## 3. Discussion

To our knowledge, this is the first description of a lupus nephritis diagnosed in the context of a renal lesion suggestive of malignancy. In the partial nephrectomy specimen, no tumorous or neoplastic cells were found but only features compatible with LN. IgG4 renal disease was considered as it may also present as inflammatory mass and membranous nephropathy, but IgG4 serum levels and immunostaining on the biopsy were not in favour of this diagnosis [2]. Renal disease in SLE is usually not associated with renal masses but, in this case, it appears it was the result of a dense lymphocytic infiltrate.

According to the Systemic Lupus International Collaborating Clinics (SLICC) group, biopsy-proven LN in presence of ANA or anti-DNA antibodies is now sufficient to make a diagnosis of SLE [3]. Renal involvement is frequent among SLE patients and presentation is highly variable [4]. True prevalence of renal-limited SLE is not well described, as this disease is usually accompanied by systemic manifestations on diagnosis or can herald the arrival of clinical SLE. However, cases of LN with negative serologies and without subsequent development of systemic involvement have been described [5].

Silent LN is usually defined as biopsy-proven LN without any renal clinical evidence of disease activity [6]. In silent LN, renal pathology usually shows isolated mesangial disease, although it can also demonstrate focal of diffuse proliferative disease. In a series of 86 SLE patients without clinical signs of renal involvement, 15 percent had class III or IV nephritis and 10 percent had class V membranous nephritis [7]. Prognosis of silent LN ranges from benign renal evolution to progression into a clinically active disease [7, 8].

This case of lupus nephritis is unusual because of its initial manifestation as a malignancy-suspected renal mass. The evolution suggests a case of early silent LN. Prevalence, prognosis, and usefulness of immunosuppressive treatment in similar cases remain unclear as this condition would usually not warrant a kidney biopsy. Further studies are needed to better characterize silent LN.

## Figures and Tables

**Table 1:** Investigation results at time of partial nephrectomy specimen and subsequent kidney biopsy after 12 months of clinical follow-up.

Investigation	Results	
	Partial nephrectomy	Biopsy
Serum creatinine	60 μmol/L	61 μmol/L
Microscopic hematuria	3–5 red blood cells per field	3–5 red blood cells per field
Urinary albumin/creatinine ratio	7.6 mg/mmol	10 mg/mmol
ANA titer	<1 : 80	<1 : 80
Anti-DNA	424 × 10^3^ IU/L	<55 × 10^3^ IU/L
Anti-SSA/Rho	10.4 IU/L	—
Anti-SSB/La	15.9 IU/L	—
C3 complement	1.22 g/L	1.08 g/L
C4 complement	0.15 g/L	0.19 g/L
Serum IgG	28.7 g/L	—
IgG4 subclass	0.6 g/L	—

## Key Message

Lupus nephritis can present itself as a solid mass suggestive of neoplasm.
